# Quantum Theory of Massless Particles in Stationary Axially Symmetric Spacetimes

**DOI:** 10.3390/e23091205

**Published:** 2021-09-13

**Authors:** Amnon Moalem, Alexander Gersten

**Affiliations:** Department of Physics, Ben Gurion University of the Negev, Beer-Sheva 84105, Israel; alex.gersten@gmail.com

**Keywords:** quantum equations, massless particles, spacetime curvature, gravity impact

## Abstract

Quantum equations for massless particles of any spin are considered in stationary uncharged axially symmetric spacetimes. It is demonstrated that up to a normalization function, the angular wave function does not depend on the metric and practically is the same as in the Minkowskian case. The radial wave functions satisfy second order nonhomogeneous differential equations with three nonhomogeneous terms, which depend in a unique way on time and space curvatures. In agreement with the principle of equivalence, these terms vanish locally, and the radial equations reduce to the same homogeneous equations as in Minkowski spacetime.

## 1. Introduction

In a sufficiently small region of spacetime, no experiment can distinguish between gravity and uniform acceleration. Known as the principle of equivalence (POE), this is a fundamental hypothesis of Einstein’s theory of general relativity (GR). GR gives a unified description of gravity as a geometric property of four-dimensional spacetime (see, for example, Refs. [1,2]). Minkowski spacetime is flat and uniform throughout, and takes no account of gravitation. It serves merely as a static background for whatever physical phenomena are present. In GR, however, the spacetime is curved, due to the presence of matter, and it is no longer a static background but actively interacts with physical systems. Instead of thinking of gravity as a real force that can be detected like other fundamental forces, in GR, gravity is a feature of the spacetime itself. Gravity is the curvature of spacetime, which affects the energy and momentum of particles. Thus, a transformation from a Cartesian coordinate system to a curved coordinate system should unveil the impacts of gravity on whatever matter or energy are present. Spacetime curvature depends on the metric, and in this sense, it represents the “gravitational” potential. Our main objective in what follows is to study how curvature of a spacetime influences the dynamics of free massless particles. To this aim, we consider quantum equations for free massless particles of any spin in a flat Minkowski spacetime and in axially symmetric spacetimes, namely, the static Schwarzschild, Friedmann–Robertson–Walker (FRW) and the stationary rotating Kerr spacetimes. The POE quoted above implies that in a sufficiently small region of any point of a curved spacetime, the mathematical representation of a physical law can be reduced to its representation in a Minkowski spacetime; therefore, the dynamics of particles in curved spacetimes and in a Minkowski spacetime must have some common features, and, whatever differences are present, they must vanish locally. The organization of this paper is as follows. In Section 2, we consider quantum equations for free massless particles of any spin in Minkowski spacetime. In Section 3, we reformulate the analogous equations in an arbitrary curved spacetime. To track the impacts of gravity in some detail, we study these equations in the axially symmetric spacetimes mentioned above in Section 4, whereby simple quantum modes are found. In turn, we use these modes to carry out quantization of the corresponding fields in Section 5. We conclude in Section 6. Some details of mathematical calculations are reserved to the Appendix A. Axially symmetric spacetimes are exact solutions of the Einstein field equations and serve as models of black holes and neutron stars. Generally speaking, we anticipate that the results we report herewith hold well for particles in the vicinity of massive objects.

Throughout this work, we use natural units with ℏ=c=1 and a metric signature (−,+,+,+). Greek indices refer to the general world index, whilst Latin indices refer to a flat Minkowskian spacetime. We take Minkowski metric to be $\;\eta_{ab}=diag\left(-1,+1,+1,+1\right)$ and $g_{\mu \nu }\left(x\right)=e_{\mu }^{a}\left(x\right)e_{\nu }^{b}\left(x\right)\eta_{ab}$, the metric of an arbitrary curved spacetime. Here, $e_{\mu }^{a}$ and $E_{a}^{\mu }$ stand for a vierbein field and its inverse, respectively (see, for example, Ref. [3] for more details). Though expected to be quantized, the spacetimes are taken to be continuous.

## 2. Field Equations in Minkowski Spacetime

Relativistic wave equations in Minkowski spacetime for free particles were obtained long ago by factorizing the Klein–Gordon operator. Dirac’s equation was derived from the relativistic condition on energy, mass and momentum. Dirac derived equations for spin *s* particles, which are written, in ordinary vector notation, as one main equation and three subsidiary conditions [4]. These four equations were derived and studied extensively by Bacry [5], using Wigner’s condition [6] on the Pauli–Lubanski Vector [7,8] for massless fields. More recently, quantum equations for free massless particles in a Minkowski spacetime were derived from first principles [9,10,11,12] by applying general methods based on the factorization of the d’Alembertian operator and from a scalar Lagrangian. Applying these methods, quantum equations for free massless particles of any spin with the subsidiary conditions included implicitly, are written as a single matrix equation in the following form:

(1) $$i\left(\Gamma_{0}^{\left(4s\right)}\partial_{t}+\Gamma^{\left(4s\right)}\cdot \nabla \right)\Phi^{\left(4s\right)}=0,$$

(2) $$\;i\left(\Gamma_{0}^{\left(4s\right)}\partial_{t}-\Gamma^{\left(4s\right)}\cdot \nabla \right)\Phi^{*\left(4s\right)}=0,$$

where $\Gamma_{i}^{\left(4s\right)}$ are 4s×4s Hermitian matrices and $\Gamma_{0}^{\left(4s\right)}$ stands for the identity matrix. These matrices form a reducible representation of the Pauli matrices and have the same eigenvalues as the Pauli matrices, i.e., ±1. Following Ref. [10], we use the angular basis of the $D^{\left(s-\frac{1}{2},\frac{1}{2}\right)}$ representation of the Lorentz group. This basis is the sum of the basis of spin *s* and s−1, altogether (2s+1)+(2s−1)=4s components. Explicitly the wave function is taken to be as follows:

(3) $$\;\Phi^{\left(4s\right)}=\left(\begin{array}{c} l\psi_{s-1}^{\left(2s-1\right)}\\ .\\ .\\ .\\ \psi_{s-1}^{\left(2s-1\right)}\\ \psi_{s}^{\left(2s+1\right)}\\ .\\ .\\ .\\ \psi_{-s}^{\left(2s+1\right)} \end{array}\right).$$

As shown in Ref. [10], the advantage of using this basis is that by equating the spin (s−1) components of the wave function $\psi_{\lambda }^{\left(2s-1\right)}$, (2s−1) subsidiary conditions which eliminate non-forward and non-backward helicities are automatically satisfied so that solutions of Equations (1) and (2) become restricted to forward and backward helicity, respectively (for more details, see Ref. [10]). The Hermitian conjugate of $\Phi^{\left(4s\right)}$ is defined as the transposed of the complex conjugate ${\left[\Phi^{\left(4s\right)}\right]}^{H}=$${\left[\Phi^{\left(4s\right)*}\right]}^{T}$ so that ${\left[\Phi^{\left(4s\right)}\right]}^{H}\Phi^{\left(4s\right)}$ is positive definite and is interpreted as a probability density. For spin 1/2 particles, Equations (1) and (2) coincide with those of left and right Majorana particles. For spin 1, they are equivalent to the four Maxwell’s equations, and for spin 2, to Dirac’s equations [4]. For the spin 1 and 2 particles, the Γ matrices are constructed to exactly reproduce the main equations and subsidiary conditions [9].

Each of Equations (1) and (2) has only two solutions corresponding to positive and negative energy. Solutions of Equation (1) are restricted to forward helicity, and those of Equation (2) to backward helicity. As the energy operator is proportional to the helicity, solutions of Equation (1) are also the solutions of Equation (2) with the opposite sign of helicity. Thus, the positive energy forward helicity solution of Equation (1) is the negative energy backward helicity solution of Equation (2); likewise, the negative energy forward helicity solution of Equation (1) is the positive energy backward helicity solution of Equation (2). Imposing the physical requirement that the energy of massless particles is restricted to positive values, a general solution of these equations can be written as a combination of positive energy solutions with opposite helicities. In the interest of generality, we replace the $\Gamma^{\mu }$ matrices with 4s×4s block diagonal matrices $\gamma^{\mu }=diag\left(\sigma^{\mu },\cdot \cdot ,\sigma^{\mu }\right)$, where $\sigma^{\mu }$(μ=0,1,2,3) denoting the Pauli matrices. The Γs are related to the $\gamma^{\mu }$ via a similarity transformation; $\gamma^{\mu }=S\Gamma^{\mu }S^{-1}$, where *S* is non-singular and unitary. With the $\gamma^{\mu }$ matrices, Equation (1) reads as follows:

(4) $$i\gamma^{b}\eta_{ab}\partial_{a}\Phi^{\left(4s\right)}=0,a=t,x,y,z,$$

with the new solutions being $\Phi_{\gamma }^{\left(4s\right)}=S\Phi_{\Gamma }^{\left(4s\right)}.$ We use the above expression as our starting point to derive particle equations in an arbitrary curved spacetime.

## 3. Field Equations in Curved Spacetime

Based on the principle of equivalence, a local Minkowski metric can be associated to every regular point (to exclude singularities) of curved spacetime. Accordingly, Equation (4) is valid within a sufficiently small region around any point of a curved spacetime. In what follows, we derive particle equations in a global curved spacetime, analogous to Equation (4). To do so, three modifications are required. First, the Minkowski metric $\eta_{ab}$ must be replaced by a global metric tensor $g_{\mu \nu }$. Secondly, the partial local derivatives $\partial_{a}$ must be replaced by covariant derivatives $\left[\partial_{\nu }+\Omega_{\nu }\left(x\right)\right]$, where $\Omega_{\nu }\left(x\right)$ is a connection component for the spinor wave function Φ. Thirdly, the local gamma matrices $\gamma^{a}$(a=t,x,y,z), which carry local Minkowski space indices must be replaced by global gamma matrices $\gamma^{\mu }=E_{a}^{\mu }\gamma^{a}$, where $E_{a}^{\mu }$ stands for inverse vierbein fields [3]. Part of the discussion to follow is reported briefly in Ref. [13]. Inserting these modifications in Equation (4) yields the following:

(5) $$E_{a}^{\mu }\gamma^{a}g_{\mu \nu }\left[\partial_{\nu }+\Omega_{\nu }\left(x\right)\right]\Phi \left(x\right)=0\;.$$

To evaluate the connection $\Omega_{\mu }\left(x\right)$, we consider the scalar $S\left(x\right)=\Phi^{H}\left(x\right)\gamma^{0}\Phi \left(x\right)$ and the vector $V^{a}\left(x\right)=\Phi^{H}\gamma^{a}\Phi \left(x\right)$. These must obey the transformation rules for scalars and vectors, respectively. A parallel transport of the wave function should satisfy the following relation:

(6) $$\Phi \left(x+dx\right)=\Phi \left(x\right)-\Omega_{\mu }\left(x\right)\Phi \left(x\right)dx^{\mu }\;.$$

Consider first S(x), which must remain unchanged under parallel transport. With Equation (6), one obtains the following:

(7) $$S\left(x+dx\right)=S\left(x\right)-\Phi^{H}\left(x\right)\left[\gamma^{0}\Phi_{\mu }^{H}\left(x\right)+\Omega_{\mu }\left(x\right)\gamma^{0}\right]\Phi \left(x\right)dx^{\mu },$$

where terms of order $dx^{\mu }dx^{\nu }$ are omitted. For Equation (7) to satisfy a scalar parallel transport rule, the quantity in the square bracket must vanish, i.e., the following:

(8) $$\Omega_{\mu }^{H}\left(x\right)=-\Omega_{\mu }\left(x\right).$$

Next, consider the vector $V^{a}\left(x\right)$, which must transport as a local vector, i.e., $V^{a}\left(x⟶x+dx\right)=V^{a}\left(x\right)-\omega_{\mu b}^{a}\left(x\right)V^{a}\left(x\right)dx^{\mu }$, where $\omega_{\mu b}^{a}\left(x\right)$ is the spin connection [3]. Again, using Equation (6), one obtains the following:

(9) $$V^{a}\left(x+dx\right)=V^{a}\left(x\right)-\Phi^{H}\left(x\right)\left[\gamma^{a},\Omega_{\mu }\left(x\right)\right]\Phi \left(x\right)dx^{\mu }.$$

Then, the second term in the above expression must satisfy the following:

(10) $$\Phi^{H}\left(x\right)\left[\gamma^{a},\Omega_{\mu }\left(x\right)\right]\Phi \left(x\right)=\omega_{\mu b}^{a}V^{b}.$$

This last expression indicates that the commutator in the expression above is proportional to $\gamma^{b}$ and that $\Omega_{\mu }$ is related to a product of two gamma matrices. Assume now that the following holds:

(11) $$\Omega_{\mu }=C\omega_{\mu bc}\gamma^{b}\gamma^{c},$$

where *C* is a constant to be determined. It is straightforward to show, using properties of the gamma matrices (see Appendix A), that the following commutator holds:

(12) $$\left[\gamma^{a},\Omega_{\mu }\left(x\right)\right]=4C\omega_{\mu b}^{a}\gamma^{b}.$$

Comparing Equation (12) with Equation (10) gives C=1/4 and the following:

(13) $$\Omega_{\mu }=\left(1/4\right)\omega_{\mu bc}\gamma^{b}\gamma^{c}.$$

Then, substitute the above result in the spin connection term in Equation (5), and using the well-known expression $\omega_{\mu b}^{a}=e_{\mu }^{a}E_{b}^{\sigma }\Gamma_{\sigma \mu }^{\nu }+e_{\nu }^{a}\partial_{\mu }E_{b}^{\nu }$ for the spin connection [3], Equation (5) becomes the following:

(14) $$E_{a}^{\mu }\gamma^{a}\Omega_{\mu }=\left(1/2\right)\gamma^{b}\left[E_{b}^{\sigma }\partial_{\sigma }lne+\partial_{\sigma }E_{b}^{\sigma }\right],$$

where $g=\det(g_{\mu \nu })$ is the determinant of the metric tensor and e=√(−g). Substituting this last expression in Equation (5) gives the following:

(15) $$E_{a}^{\mu }\gamma^{a}\partial_{\mu }\left(\surd e\Phi \right)=-1/2\gamma^{b}\partial_{\sigma }E_{b}^{\sigma }\Phi .$$

It is straightforward to verify that the expression above can be derived from the Lagrangian density, as follows:

(16) $$L=\Phi^{H}\left(E_{a}^{\mu }\gamma^{a}\partial_{\mu }\left(\sqrt{e}\Phi \right)+\frac{1}{2}\gamma^{b}\partial_{\sigma }E_{b}^{\sigma }\Phi \right).$$

The variation with respect to $\Phi^{H}$ obviously yields Equation (15), while variation with respect to Φ gives the Hermitian conjugate equation, i.e., the following:

(17) $$E_{a}^{\mu }\gamma^{a}\partial_{\mu }\left(\sqrt{e}\Phi^{*}\right)=-\frac{1}{2}\gamma^{b}\partial_{\sigma }E_{b}^{\sigma }\Phi^{*}.$$

Equations Equations (15) and (17) replace the Minkowskian particle equations of Equation (4) and its Hermitian conjugate. These are generic equations that are valid for any curved spacetime and meet well with the POE. It is easy to see this, using different wording of the POE; at every point of a spacetime, the metric can be diagonalized as $g_{ij}=\left(-1,1,1,1\right).$ Clearly substituting this for any metric, Equations (15) and (17) reduce rigorously to the analogous Minkowskian equations.

From the Lagrangian Equation (16) and using Noether’s theorem, the conserved current density is given by the following:

(18) $$j^{\mu }=\frac{\delta L}{\delta \left(\partial_{\mu }\Phi_{b}\right)}\frac{\delta \Phi^{b}}{\delta \alpha }-J_{0}^{\mu }=\Phi_{b}^{H}E_{a}^{\mu }\gamma^{a}\Phi^{b}=\Phi^{H}\gamma^{\mu }\Phi .$$

Note that $j^{0}=\Phi^{H}\gamma^{0}\Phi =\Phi_{b}^{*}\Phi^{b}$ is a probability density. The expression above satisfies the continuity equation, i.e., the following:

(19) $$\partial_{\mu }j^{\mu }=\partial_{\mu }\left[\Phi^{H}\gamma^{\mu }\Phi \right],$$

and does not depend on the spin connection. As shown in the Appendix A, a simple calculation based on Equation (8) explains why the conserved current does not depend on the spin connection.

## 4. Factorization of Φ

To make any further progress using Equation (15), a specific choice of a metric tensor must be made. To analyze axially symmetric spacetimes, we apply a procedure based on the factorization of the wave function Φ. We assume that the wave function Φ factorizes as follows:

(20) $$\Phi \left(t,r,\theta ,\varphi \right)=\psi \left(t,r,\theta ,\varphi \right)f\left(r,\theta \right),$$

where the function ψ satisfies an equation independent of the spin connection, i.e., the following:

(21) $$E_{a}^{\mu }\gamma^{a}\partial_{\mu }\psi \left(t,r,\theta ,\varphi \right)=0.$$

In what follows, we refer to this equation as the reduced equation and to its solutions as the reduced wave function. A similar factorization is applied in Refs. [14,15] to deal with Dirac’s equation. Note that for the Minkowski, Schwarzschild and FRW spacetimes equation, Equation (21) involves diagonal metric elements. For the Kerr metric equation, Equation (21) involves also two non-diagonal metric elements, namely $E_{0}^{3}$ and $E_{3}^{0}.$ To treat all cases on an equal basis, we eliminate these non-diagonal elements by requiring a second factorization as described below. Inserting Equations (20) and (21) in Equation (15), one finds that the factor $f\left(r,\theta \right)$ satisfies the following equation:

(22) $$\partial_{\mu }\ln\left(f\left(r,\theta \right)\sqrt{e}\right)=-\frac{1}{2}e_{\mu }^{b}\partial_{\sigma }E_{b}^{\sigma }.$$

Note that all complexities due to the spin connection are now restricted to the above expression. Specifically for stationary axially symmetric spacetimes, $f\left(r,\theta \right)$ does not depend on time nor on the azimuthal coordinates, and Equation (22) becomes rather simple as follows:

(23) $$\partial_{\mu }\ln\left(f\left(r,\theta \right)\sqrt{e}\right)=-\frac{1}{2}e_{\mu }^{b}\partial_{\sigma }E_{b}^{\sigma },b=1,2.$$

For the Minkowski, Schwarzschild, FRW and Kerr metrics (see Table 1), $\partial_{r}\ln\left(f\left(r,\theta \right)\sqrt{e}\right)=-\frac{1}{2}e_{1}^{1}\partial_{r}E_{1}^{1}$ and $\partial_{t}\ln\left(f\left(r,\theta \right)\sqrt{e}\right)=\partial_{\theta }\ln\left(f\left(r,\theta \right)\sqrt{e}\right)=\partial_{\varphi }\ln\left(f\left(r,\theta \right)\sqrt{e}\right)=0$. Thus, Equation (22) is integrable, giving rise to the simple analytic form of $f\left(r,\theta \right)$ listed in Table 1. The idea behind the factorization Equation (20) is to bring the reduced equation, Equation (21), as close as possible to the equation in a Minkowski spacetime. Following the factorization Equation (20), this is well accomplished for the Schwarzschild and FRW metrics. As indicated above for the Kerr metric the resulting reduced equation following this first factorization still involves the non-diagonal vierbein elements $E_{0}^{3}$ and $E_{3}^{0}$. To eliminate these, we apply two consecutive factorizations. As a first step, take ϕ to be the reduced function following first factorization, and solve for $f\left(r,\theta \right)$. Using the vierbein field listed in Table 1, one finds that the determinant of the vierbein $e=\rho^{2}\sin\theta ,\;\partial_{t}\log\left[h\right]=-\frac{1}{2}E_{b}^{\lambda }\partial_{\lambda }e_{0}^{b}=0,$$\partial_{\varphi }\log\left[h\right]=-\frac{1}{2}E_{b}^{\lambda }\partial_{\lambda }e_{\varphi }^{b}=0$, $\partial_{\theta }\log\left[h\right]=-\frac{1}{2}E_{2}^{2}\partial_{\theta }e_{2}^{2}=0,$ and $\partial_{\rho }\log\left[h\right]=\partial_{\rho }\log{\left(\frac{\rho }{\sqrt{\Delta }}\right)}^{-1/2}$, where $f\left(r,\theta \right)=he^{1/2}$. This yields the following:

(24) $$f\left(r,\theta \right)=he^{1/2}={\left(\frac{\sqrt{\Delta }}{\rho }\right)}^{1/2}\rho \sin^{1/2}\theta$$

Following this step, the equation for ϕ reads as follows:

(25) $$\left[E_{0}^{0}\gamma^{0}\partial_{t}+E_{3}^{0}\gamma^{3}\partial_{t}+E_{1}^{1}\gamma^{1}\partial_{r}+E_{2}^{2}\gamma^{2}\partial_{\theta }+E_{0}^{3}\gamma^{0}\partial_{3}+E_{3}^{3}\gamma^{3}\partial_{3}\right]\phi =0.$$

In order to eliminate the two terms which involve the non-diagonal vierbein, we assume additional factorization of ϕ,$\phi \left(t,r,\theta ,\varphi \right)=g\left(\theta ,\varphi \right)\psi \left(t,r,\theta ,\varphi \right)\;$ and require that ψ satisfies an equation, which involves diagonal metric elements only, i.e., the following:

(26) $$\left[E_{0}^{0}\gamma^{0}\partial_{t}+E_{1}^{1}\gamma^{1}\partial_{r}+E_{2}^{2}\gamma^{2}\partial_{\theta }+E_{3}^{3}\gamma^{3}\partial_{3}\right]\psi =0.$$

This coincides with Equation (21) for the other cases. Substituting the above equation in Equation (25) and taking $\psi ∼\exp\left(-i\omega t\right)\exp\left(im\varphi \right)$ gives the following expression for $g\left(\theta ,\varphi \right)$:

(27) $$E_{1}^{1}\gamma^{1}\partial_{r}\left(\ln g\right)+E_{2}^{2}\gamma^{2}\partial_{\theta }\left(\ln g\right)=i\left[\omega E_{3}^{0}\gamma^{3}-mE_{0}^{3}\gamma^{0}\right].$$

To evaluate $g\left(\theta ,\varphi \right)$, we must first eliminate the gamma matrices in the above expression. To this aim, we square the left and right sides of the above equation and then take their traces. Using properties of the gamma matrices (see Appendix A) one obtains the following:

(28) $${\left[E_{1}^{1}\partial_{r}\ln g\left(\theta ,\varphi \right)\right]}^{2}+{\left[E_{2}^{2}\partial_{\theta }\left(\ln g\left(\theta ,\varphi \right)\right)\right]}^{2}=-\left[{\left(\omega E_{3}^{0}\right)}^{2}+{\left(mE_{0}^{3}\right)}^{2}\right].$$

Finally assuming g(r,θ)=A(r)B(θ) and substituting the inverse vierbein from Table 1, the expression above is equivalent to a pair of integrable equations as follows:

(29) $$\partial_{r}\left(\ln A(r)\right)=ima,$$

and,

(30) $$\partial_{\theta }\left(\ln B(\theta )\right)=i\omega a\sin\theta .$$

These give,

(31) $$g\left(r,\theta \right)=A\left(r\right)B\left(\theta \right)=\exp ia\left(mr-\omega \cos\theta \right).$$

With this accomplished, the wave function $\Phi \left(t,r,\theta ,\varphi \right)=g\left(r,\theta \right)f\left(r,\theta \right)\psi \left(t,r,\theta ,\varphi \right),$ where the reduced function ψ satisfies the following equation:

(32) $$\left[\frac{1}{\sqrt{\Delta }\rho }\left(a^{2}+r^{2}\right)\gamma^{0}\partial_{0}+\frac{\sqrt{\Delta }}{\rho }\gamma^{1}\partial_{1}+\frac{1}{\rho }\gamma^{2}\partial_{2}+\frac{1}{\rho \sin\theta }\gamma^{3}\partial_{3}\right]\psi \left(t,r,\theta ,\varphi \right)=0.$$

Note that the factor $\left(1/\rho \right)$ in the above equation can be eliminated, and we may conclude that the reduced function ψ in Kerr spacetime satisfies Equation (21), where only diagonal metric elements are involved, just as for the other cases. Moreover, the last two terms in Equation (21), which determine the reduced angular wave function, are common to all of the Schwarzschild, FRW, Kerr and Minkowski spacetimes. Thus, the reduced angular wave functions for the Schwarzschild, FRW and Kerr metrics are the same as in the Minkowskian case, and the impacts of gravity, whatever they may be, are due to $E_{0}^{0}$ and $E_{1}^{1}$.

### 4.1. The Reduced Wave Function ψ

It is instructive to consider first Equation (15) in the Minkowskian case, where the metric, $g_{\mu \nu }=diag\left(1,-1,-1,-1\right)$ and $e=\sqrt{-\det g}=1.$ The procedure to be applied for other cases is rather similar and, as indicated already, the reduced angular wave function is the same. With $\Phi ∼\exp\left(-i\omega t\right)\exp\left(im\varphi \right),$ Equations (16) and (17) reduce to the following:

(33) $$\omega E_{0}^{0}\Phi =-i\gamma \cdot \nabla \Phi =H_{0}\Phi ,$$

(34) $$\omega E_{0}^{0}\Phi^{*}=i\gamma \cdot \nabla \Phi^{*}=H_{0}\Phi^{*}.$$

Here, **∇** is the Del. operator, and $H_{0}=-i$ γ·∇ is the Hamiltonian of the unperturbed free states. Naturally, these equations do not depend on the spin connection. In spherical coordinates, we have the following:

(35) $$\nabla =\hat{r}\;\partial_{r}+\hat{\theta }\frac{1}{r}\;\partial_{\theta }+\hat{\phi }\frac{1}{r\sin\theta }\;\partial_{\varphi },$$

and the free Hamiltonian reads as follows:

(36) $$H_{0}=-i\gamma \;\cdot \nabla =-i\gamma^{r}\partial_{r}+\gamma^{\theta }\frac{1}{r}\;\partial_{\theta }+\gamma^{\varphi }\;\frac{1}{r\sin\theta }\partial_{\varphi },$$

where the matrices $\gamma^{t},\gamma^{r},\gamma^{\theta },\gamma^{\varphi }$ are given via the following transformation:

$$\left(\begin{array}{c} \gamma^{t}\\ \gamma^{r}\\ \gamma^{\theta }\\ \gamma^{\varphi } \end{array}\right)=\left(\begin{array}{cccc} E_{0}^{0} & 0 & 0 & E_{0}^{3}\\ 0 & E_{1}^{1} & 0 & 0\\ 0 & 0 & E_{2}^{2} & 0\\ E_{3}^{0} & 0 & 0 & E_{3}^{3} \end{array}\right)\left(\begin{array}{c} \gamma^{0}\\ \gamma^{1}\\ \gamma^{2}\\ \gamma^{3} \end{array}\right).$$

Again, the inverse vierbein fields $E_{2}^{2}=1/r$ and $E_{3}^{3}=1/r\sin\theta$ are common to the axially symmetric spacetimes considered. This ascertains that the reduced angular wave function is the same as in the Minkowskian case. This is derived rigorously below. However, the inverse vierbein fields $E_{0}^{0}$ and $E_{1}^{1}$ are signatures of a metric giving rise to different reduced radial wave functions for each case.

#### 4.1.1. The Reduced Angular Wave Function

By definition, the angular momentum operator is defined as $\hat{L}=-i\hat{r}\times \nabla$. Then, using Equation (35), one obtains the following:

$$\hat{r}\times \hat{L}=i\left(\hat{\theta }\partial_{\theta }+\hat{\phi }\;\partial_{\phi }\right),$$

and

(37) $$\gamma \;\cdot \hat{r}\times L=\frac{1}{r}i\left(\gamma^{\theta }\partial_{\theta }+\gamma^{\phi }\partial_{\phi }\right)=i\frac{1}{r}\left(E_{2}^{2}\gamma^{2}\partial_{\theta }+E_{3}^{3}\gamma^{3}\partial_{\phi }\right).$$

We now use this expression to define an angular operator *K*. Based on the properties of the gamma matrices, any three vectors A and B satisfy the following identity (see Appendix A):

(38) $$\left(\gamma^{\;}\cdot A\right)\left(\gamma^{\;}\cdot B\right)=\gamma^{0}A\cdot B+\frac{i}{2}\gamma \cdot \left(A\times B\right).$$

Then, for $A=\hat{r}\;$ and B=L,, one obtains the following:

(39) $$\;\gamma^{\;}\cdot \hat{r}\;\times L=-i\frac{1}{r}\left(\gamma \cdot \hat{r}\right)\left(2\gamma^{\;}\cdot L\right).$$

With this relation, the free particle Hamiltonian reads as follows:

(40) $$H_{0}=-i\gamma \cdot \nabla =-i\gamma \cdot \hat{r}\;\left[\partial_{r}-\frac{1}{r}\;\left(2\gamma \cdot L\right)\right].$$

Finally, we identify the spin S with γ and define an angular operator *K* as follows:

(41) $$K=2\;\gamma \cdot L+s=2S\cdot L+s=J^{2}-L^{2}-S^{2}+s,$$

where L,S, and J=L+S are, respectively, the orbital, spin, total angular momenta and s is the particle spin. Substituting this in Equation (40) yields the following:

(42) $$H_{0}=-i\gamma \cdot \hat{r}\left[\partial_{r}+\frac{s}{r}-\frac{1}{r}K\right].$$

This expression of the free Hamiltonian suggests that we may construct the solution Φ by variable separation. Indeed, $H_{0},J^{2},J_{3},K$ and parity P constitute a complete set of commuting operators (see Appendix A). The angular part of the wave function Φ can be readily written as a spinor of spherical harmonics, i.e., the following:

(43) $$Y_{lm_{j}}^{j}\left(\Omega \right)=\sum_{m_{s}=-s,s}C\left(l,s,j;m_{j}-m_{s},m_{s},m_{j}\right)Y_{l}^{m_{l}}\left(\theta ,\varphi \right)\chi_{s}^{m_{s}}.$$

Here, $C\left(l,s,j;m_{j}-m_{s},m_{s},m_{j}\right)$ is a Clebsch–Gordan coefficient for combining orbital angular momentum *l* and spin *s* to a total angular momentum *j* with magnetic quantum numbers $m_{j}-m_{s},m_{s},m_{j}$, respectively, and $\;Y_{l}^{m_{l}}\left(\theta ,\varphi \right)$ are the usual spherical harmonics, which are eigenfunctions of $L^{2},L_{3},$, and $\chi_{s}^{m_{s}}$ are eigenfunctions of $S^{2}$ and $S_{3}.$ For massless particles, $m_{s}$ can have only two values, *s* and −s. The spherical harmonic spinors are orthonormal, i.e., as follows:

(44) $$\int d\Omega {\left(Y_{l^{\prime }m_{j^{\prime }}}^{j^{\prime }}\left(\Omega \right)\right)}^{H}Y_{lm_{j}}^{j}\left(\Omega \right)=\delta_{j^{\prime }j}\delta_{ll^{\prime }}\delta_{m_{j^{\prime }}m_{j}}.$$

Furthermore, the function Φ is an eigenfunction of the operators $J^{2}$, $J_{3}$, the angular operator *K* and parity P, i.e., the following:

(45) $$J^{2}\Phi_{m_{j},\kappa }^{j}=j\left(j+1\right)\Phi_{m_{j},\kappa_{j}}^{j},$$

(46) $$J_{3}\Phi_{m_{j},\kappa }^{j}=m_{j}\Phi_{m_{j},\kappa_{j}}^{j},$$

(47) $$K\Phi_{m_{j},\kappa_{j}}^{j}=\kappa_{j}\Phi_{m_{j},\kappa_{j}}^{j}.$$

To calculate the eigenvalues values of the angular operator *K* for j=l±s, we look for eigenvalues values of the operator $K^{2}={\left(2L\cdot S+s\right)}^{2}$. From the addition of angular momenta, one finds the following:

(48) $${\left(\kappa_{j}\right)}^{2}={\left(J^{2}-L^{2}-S^{2}+s\right)}^{2}=s^{2}{\left(2l+1\right)}^{2}.$$

So that for both j=l+s and j=l−s, the eigenvalues of *K* are $\kappa_{j}=\pm s\left(2l+1\right)$. This means that for each of the cases j=l+s and j=l−s, there exist two solutions $R_{1}\left(r\right)\phi^{+}\left(\theta ,\varphi \right)$ and $R_{2}\left(r\right)\phi^{-}\left(\theta ,\varphi \right)$ corresponding to positive and negative $\kappa_{j}$, respectively. The eigenvalues of *K* span all values $-s\left(2l+1\right),-s\left(2l+1\right)+1,\cdot \cdot \cdot ,$$s\left(2l+1\right)-1,s\left(2l+1\right).$

#### 4.1.2. The Reduced Radial Wave Equation

We may now turn to consider the radial function. With the free Hamiltonian Equation (42), we write Equation (21) as follows:

$$iE_{0}^{0}\omega \psi =E_{1}^{1}\gamma^{1}\;\left[\partial_{r}+\frac{s}{r}-\frac{1}{r}K\right]\psi .$$

Then, substituting $\gamma^{\mu }=diag\left(\sigma^{\mu },\cdot \cdot \cdot ,\sigma^{\mu }\right)$, the above expression yields linear non-autonomous equations as follows:

(49) $$iE_{0}^{0}\omega I^{2}\psi =E_{1}^{1}\sigma^{1}\left[\partial_{r}+\frac{s}{r}-\frac{1}{r}K\right]\psi .$$

Then, substituting $\psi ∼\exp\left(-i\omega t\right)\exp\left(im_{j}\varphi \right)\left(\begin{array}{c} R_{1}\left(r\right)\phi^{+}\left(\theta ,\varphi \right)\\ R_{2}\left(r\right)\phi^{-}\left(\theta ,\varphi \right) \end{array}\right)$ with $K\Phi =\kappa_{j}\left(\begin{array}{c} R_{1}\left(r\right)\phi^{+}\left(\theta ,\varphi \right)\\ -R_{2}\left(r\right)\phi^{-}\left(\theta ,\varphi \right) \end{array}\right)\;$ leads to the following pair of equations:

(50) $$i\omega E_{0}^{0}R_{1}\phi^{+}=E_{1}^{1}\left[\partial_{r}+\frac{s+\kappa }{r}\right]R_{2}\phi^{-},$$

(51) $$i\omega E_{0}^{0}R_{2}\phi^{-}=E_{1}^{1}\left[\partial_{r}+\frac{s-\kappa }{r}\right]R_{1}\phi^{+}.$$

We may rewrite these as autonomous equations for the radial functions $R_{1}\left(r\right)$ and $R_{2}\left(r\right)$, i.e., the following:

$$\frac{d^{2}R_{1}}{dr^{2}}+2\frac{s}{r}\frac{dR_{1}}{dr}+\left[\frac{1}{r^{2}}\left[\left(s\left(s-1\right)-\left(\kappa -1\right)\kappa \right)\right]+\omega^{2}\right]R_{1}\left(r\right)$$

(52) $$=\omega^{2}\left[1-{\left(\frac{E_{0}^{0}}{E_{1}^{1}}\right)}^{2}\right]R_{1}-\frac{d\ln\left(\frac{E_{1}^{1}}{E_{0}^{0}}\right)}{dr}\frac{s-\kappa }{r}R_{1}-\frac{d\ln\left(\frac{E_{1}^{1}}{E_{0}^{0}}\right)}{dr}\frac{dR_{1}}{dr},$$

and,

$$\frac{d^{2}R_{2}}{dr^{2}}+2\frac{s}{r}\frac{dR_{2}}{dr}+\left[\frac{1}{r^{2}}\left[\left(s\left(s-1\right)-\kappa \left(\kappa +1\right)\right)\right]+\omega^{2}\right]R_{2}\left(r\right)=$$

(53) $$\omega^{2}\left[1-{\left(\frac{E_{0}^{0}}{E_{1}^{1}}\right)}^{2}\right]R_{2}\left(r\right)-\frac{d\ln\left(\frac{E_{1}^{1}}{E_{0}^{0}}\right)}{dr}\frac{s+\kappa }{r}R_{2}-\frac{d\ln\left(\frac{E_{1}^{1}}{E_{0}^{0}}\right)}{dr}\frac{dR_{2}}{dr}\;.$$

In the generic expressions above, the nonhomogeneous terms depend, in a unique way, on time curvature ($E_{0}^{0}$) and space curvature ($E_{1}^{1}$), representing the impact of gravity and the coupling of the gravitational field to all other fields. Of course, the curvature of a spacetime depends on the metric. The curvature is created by energy and momentum and must be different in the case of the stationary rotating Kerr spacetime from that of the static Schwarzschild spacetime. For example, the quantity $\left(E_{0}^{0}/E_{1}^{1}\right)=\left(a^{2}+r^{2}\right)/\sqrt{\Delta }$ depends on rotation energy; energy, like matter, contributes to the spacetime curvature. Turning off this energy by setting the angular momentum to be zero (a=0) (see Table 1), and taking $r_{s}=2GM$, the nonhomogeneous terms reduce, as expected, to those of the static non-rotating Schwarzschild spacetime. What is also true is that, with a=0, the normalization function N(r,θ) becomes the same. The nonhomogeneous terms tell how gravity affects the physics of massless particles. Obviously, these terms vanish in the Minkowskian case, and the radial equations above become homogeneous. It is straightforward to show also that these terms vanish locally. Again, as suggested by the POE at every point of spacetime, the metric can be diagonalized as $g_{ij}=\left(-1,1,1,1\right)$ so that the equations above indeed reduce locally to the Minkowskian homogeneous equations for all the spacetimes considered.

It is worth noting that by replacing κ by −κ in the equation for $R_{2}/R_{1}$, we obtain the equation for $R_{1}/R_{2}.$ It is also worth mentioning that in the Minkowskian case, the equations above for spin s=1 particles are the familiar spherical Bessel’s equations, and their solutions are the spherical Bessel’s functions $j_{\kappa }\left(r\right)$ with κ=1,3,5,···· for $R_{1}\left(r\right)$ and $j_{-k-1}\left(r\right)$ with −κ−1=−2,−4,−6,··· for $R_{2}\left(r\right).$

### 4.2. Quantum Modes of Massless Particles

To conclude this section, let us summarize the main results. We have demonstrated that the wave function Φ can be factorized into a normalization function N(r,θ) and a reduced function ψ, which depends on diagonal elements of the metric. In particular, the elements $E_{2}^{2}$ and $E_{3}^{3}$ are practically the same as in the Minkowskian case; therefore, the reduced angular wave functions are spherical harmonic spinors corresponding to simple quantum modes, which we may specify by the particle orbital angular momentum $\left(l=0,1,2,\cdot \cdot \cdot \right)$ and its z components $,\left(m_{l}=-l,-l+1\cdot \cdot \cdot \cdot ,l-1,l\right),$ the spin *s* and its z components $\left(s\;,\;m_{s}=-s,s\right),$ total angular momentum $\left(j=l+s\right)$ and its z components $\left(m_{j}=-j,\cdot \cdot \cdot ,j\right)$, and the eigenvalues $\kappa_{j}=\pm s\left(2l+1\right)$ of the angular operator *K*. Quantum modes of free massless particles of any spin in any of the spacetimes considered share the above quantum numbers. However, the radial functions are signatures of a metric, and the radial quantum number of these modes is expected to be different.

## 5. Quantization

We may now use the quantum modes found in the previous section for canonical quantization of the wave functions.

### 5.1. Second Quantization of Φ

The solutions of Equations (33) and (34) correspond, respectively, to forward and backward helicity. Each of these has two energy solutions, one positive and one negative, where the negative energy forward helicity solution of Equation (33) is identical to the positive energy backward solution of Equation (34); likewise, the negative backward solution of Equation (34) is the same as the positive energy forward helicity solution of Equation (33). Restricting the energy of massless particles to be positive, we expand the wave function Φ in terms of positive energy eigenstates. Let $u\left(p,s\right)\exp\left(-ipx\right)$ denote a positive energy forward helicity state, and let $v\left(p,s\right)\exp\left(ipx\right)$ denote a positive energy backward helicity state. Inserting these into Equations (33) and (34) gives the following:

(54) $$\left(\omega_{p}E_{0}^{0}-\gamma \cdot p\right)u\left(p\right)=0,\;\;\;\mathrm{and}\;\;\;\left(\omega_{p}E_{0}^{0}+\gamma \cdot p\right)v\left(p\right)=0.$$

Here, $\omega_{p}=p_{0}=\pm \sqrt{p\cdot p}$. The spinors $u\left(p\right)$ and $v\left(p\right)$ are orthogonal so that the following holds:

(55) $$u{\left(p\right)}^{H}v\left(p\right)=v^{H}\left(p\right)u\left(p\right)=0.$$

We normalize them according to the following:

(56) $$u^{a}\left(p\right)u_{a}^{†}\left(p^{\prime }\right)=\omega_{p}\;\;;\;\;\;v^{a}\left(p\right)v_{a}^{†}\left(p^{\prime }\right)=\omega_{p}.$$

We may then expand the wave function as follows:

(57) $$\Phi \left(x\right)=\int \frac{d^{3}p}{{\left(2\pi \right)}^{3/2}}\sqrt{\frac{1}{E_{0}^{0}\omega_{p}}}\left[b\left(p\right)u\left(p\right)\exp\left(-ipx\right)+d^{†}\left(p\right)v\left(p\right)\exp\left(ipx\right)\right],$$

(58) $$\Phi^{H}\left(x\right)=\int \frac{d^{3}p}{{\left(2\pi \right)}^{3/2}}\sqrt{\frac{1}{E_{0}^{0}\omega_{p}}}\left[b^{†}\left(p\right)u^{†}\left(p\right)\exp\left(ipx\right)+d\left(p\right)v^{†}\left(p\right)\exp\left(-ipx\right)\right],$$

where $b\left(p\right)$ and $b^{†}\left(p\right)$ are annihilation and creation operators for a positive energy forward helicity, and $d\left(p\right)$ and $d^{†}\left(p\right)$ are annihilation and creation operators for a positive energy backward helicity. In order to quantize Φ, we impose the following commutators:

$$\left[\Phi \left(x\right),\Phi {\left(x^{\prime }\right)}^{H}\right]=i\delta^{3}\left(x-x^{\prime }\right),$$

$$\left[\Phi \left(x\right),\Phi \left(x^{\prime }\right)\right]=0,$$

(59) $$\left[\Phi^{H}\left(x\right),\Phi^{H}\left(x^{\prime }\right)\right]=0.$$

These are equivalent to the following commutators of creation and annihilation operators:

(60) $$\left[b\left(p\right),b^{†}\left(p^{\prime }\right)\right]=\left[d\left(p\right),d^{†}\left(p^{\prime }\right)\right]=\delta_{pp^{\prime }}\delta_{\sigma \sigma^{\prime }},$$

(61) $$\left[b^{†}\left(p\right),b^{†}\left(p^{\prime }\right)\right]=\left[d^{†}\left(p\right),d^{†}\left(p^{\prime }\right)\right]=\left[b\left(p\right),b\left(p^{\prime }\right)\right]=\left[d\left(p\right),d\left(p^{\prime }\right)\right]=0,$$

(62) $$\left[b\left(p\right),b^{†}\left(p^{\prime }\right)\right]=\left[d\left(p\right),b^{†}\left(p^{\prime }\right)\right]=\left[d^{†}\left(p\right),b\left(p^{\prime }\right)\right]=0,$$

where σ stands for helicity. This can be verified rather easily by inserting Equations (57) and (58) in Equation (59). Inserting in the double integral obtained, the normalization conditions Equation (56), the orthogonality conditions Equation (55), the commutators Equations (60)–(62), and the commutators Equation (59) are recovered.

### 5.2. Quantization of the Reduced Function ψ

Quantization of the reduced function ψ can be performed following the same procedure as applied above for Φ in the Minkowskian case. In fact, the reduced Equation (21) of the function ψ and its complex conjugate are quite similar to Equations (33) and (34):

(63) $$iE_{0}^{0}\partial_{t}\psi =-i\gamma \cdot \nabla \psi ,$$

(64) $$iE_{0}^{0}\partial_{t}\psi^{*}=i\gamma \cdot \nabla \psi^{*}.$$

Then we may expand ψ as follows:

(65) $$\psi \left(x\right)=\int \frac{d^{3}p}{{\left(2\pi \right)}^{3/2}}\sqrt{\frac{1}{E_{0}^{0}\omega_{p}}}\left[b\left(p\right)\tilde{u}\left(p\right)\exp\left(-ipx\right)+d^{†}\left(p\right)\tilde{v}\left(p\right)\exp\left(ipx\right)\right],$$

(66) $$\psi^{H}\left(x\right)=\int \frac{d^{3}p}{{\left(2\pi \right)}^{3/2}}\sqrt{\frac{1}{E_{0}^{0}\omega_{p}}}\left[b^{†}\left(p\right){\tilde{u}}^{†}\left(p\right)\exp\left(ipx\right)+d\left(p\right){\tilde{v}}^{†}\left(p\right)\exp\left(-ipx\right)\right].$$

With the spinors normalized as follows:

(67) $${\tilde{u}}^{a}\left(p\right){\tilde{u}}_{a}^{†}\left(p^{\prime }\right)=\omega_{p}/N\left(r,\theta \right)\;\;;\;\;\;{\tilde{v}}^{a}\left(p\right){\tilde{v}}_{a}^{†}\left(p^{\prime }\right)=\omega_{p}/N\left(r,\theta \right),$$

and the commutator Equations (60)–(62), we ascertain that the reduced function ψ satisfies the commutators as follows:

$$\left[\psi \left(x\right),\psi {\left(x^{\prime }\right)}^{H}\right]=i\delta^{3}\left(x-x^{\prime }\right),$$

$$\left[\psi \left(x\right),\psi \left(x^{\prime }\right)\right]=0,$$

(68) $$\left[\psi^{H}\left(x\right),\psi^{H}\left(x^{\prime }\right)\right]=0.$$

These are the same as those obtained for the function Φ Equation (59). Here as well, the role played by the function $N\left(r,\theta \right)$ is that of a normalizing factor.

### 5.3. Vacuum Energy

Let us first evaluate the vacuum energy in a Minkowski spacetime. From the Lagrangian Equation (16) and Equations (57) and (58), the Hamiltonian density and Hamiltonian are given by the following:

(69) $$H=\Phi^{H}E_{0}^{0}\omega_{k}\Phi ,$$

(70) $$H=\int d^{3}x\Phi^{H}E_{0}^{0}\omega_{k}\Phi =\int \frac{d^{3}p}{{\left(2\pi \right)}^{3}}\omega_{k}\left[b^{†}\left(p\right)b\left(p^{\prime }\right)+d\left(p\right)d^{†}\left(p\right)\right],$$

where we have used the orthogonality and normalization conditions Equations (55) and (56). With the vacuum state denoted by $|0\rangle$ and using the familiar matrix elements $\langle 0\left|b^{†}\left(p\right)b\left(p^{\prime }\right)\right|0\rangle =0,\langle 0\left|d\left(p\right)d^{†}\left(p\right)\right|0\rangle =1$, we obtain the following:

(71) $$\langle 0\left|H\right|0\rangle =\frac{V}{{\left(2\pi \right)}^{3}}\int d^{3}p\omega_{k}\to \sum_{k}\omega_{k},$$

where *V* denotes the volume of space. This same results are obtained by substituting $\Phi \left(x\right)=N$$\left(x_{1},x_{2}\right)\psi \left(x\right)$ in Equation (70) with $\psi \left(x\right)$ and $\psi^{H}\left(x\right)$ taken to be as in Equations (65) and (66), and using the normalization conditions of Equation (67). The factors $E_{0}^{0}\left(x\right)$ and $N^{H}\left(x_{1,}x_{2}\right)N\left(x_{1,}x_{2}\right)$ cancel out in any case so that Equation (71) represents the vacuum energy for all of the Minkowskian, Schwarzschild, FRW and Kerr metrics.

## 6. Summary and Discussion

We have considered quantum equations of free massless particles of any spin in a flat Minkowski spacetime and in curved spacetimes in an attempt to unveil how the space curvature affects the physics of these particles. Among these particles are the gauge bosons; the photon and gluons (spin 1) and the graviton (spin 2) are presumably carriers of fundamental interactions. A particle state is taken to be a spinor, which satisfies the generic Equation (4) in Minkowski spacetime and Equation (15) in global curved spacetime. Curvatures depend on the metric, and in order to make further progress using Equation (15), we have limited our discussion to axially symmetric spacetime. Symmetry places restrictions on the metric and allows one to employ a systematic procedure and draw some general conclusions for this group of spacetimes. Generally speaking, Equation (15) is replaced by two mathematically manageable equations, Equation (21) for the reduced function ψ and Equation (22) for the normalization function N(r,θ). Formally, the equation for ψ involves diagonal metric elements, leading to the conclusion that up to the normalization function N(r,θ), the reduced angular wave function is the same as in the Minkowskian case. The reduced radial equations, however, are found to be nonhomogeneous second order differential equations with nonhomogeneous terms, which depend on the characteristic time ($E_{0}^{0}$) and space ($E_{1}^{1}$) curvatures. The impacts of gravity are restricted to these terms. In keeping with the POE, though expressed in a unique way, these terms vanish locally, resulting in the characteristic homogeneous 2nd order differential equations of the Minkowskian case. Assuming, as in GR, that the metric of a spacetime is the “gravitation potential”, these nonhomogeneous terms are specific for a metric and can be different for different metrics. Yet, in any case for the stationary axially symmetric metrics considered, gravity affects the radial wave function only and does not affect the angular wave function.

In order to quantize Φ, we used forward and backward helicity positive energy modes. In terms of these, suitably normalized, we have demonstrated that N(r,θ) plays the role of a normalization function, which does not affect the quantization of Φ nor of ψ. This we consider to be further support to the claim that free massless particles of any spin living on curved axially symmetric spacetimes exhibit simple common features and can be studied using consistently the procedure we have applied. We believe that our work can be extended to include other metrics, such as the Reissner–Nordstrom and Kerr–Newman metrics.

Finally, axially symmetric spacetimes are models of massive objects. We anticipate that the analyses presented can also be applied for massless particles in the vicinity of black holes and neutron stars.

## Figures and Tables

**Table 1:** The Function $N\left(r,\theta \right)$ for different metrics.

Spacetime	Inverse vierbein $E_{\nu }^{\mu }$	N(r,θ) ${}^{\left(1\right)}$
Minkowski	$diag\left(1,1,\frac{1}{r},\frac{1}{\left(r\sin\theta \right)}\right)$	$r\sin^{1/2}\theta$
FRW ${}^{\left(2\right)}$	$diag\left(1,\frac{F}{a},\frac{1}{ar},\frac{1}{\left(ar\sin\theta \right)}\right)$	${\left(a^{3}r\sin\theta \right)}^{1/2}$
Schwarzschild ${}^{\left(3\right)}$	$diag\left(\frac{1}{\sqrt{F}},\sqrt{F},\frac{1}{r},\frac{1}{\left(ar\sin\theta \right)}\right)$	$F^{1/4}r\sin^{1/2}\theta$
Kerr ${}^{\left(4\right)}$	$E_{0}^{0}=\frac{\left(a^{2}+r^{2}\right)}{\sqrt{\Delta }\rho },E_{1}^{1}=\frac{\sqrt{\Delta }}{\rho },E_{2}^{2}=\frac{1}{\rho }$	
	$E_{3}^{3}=\frac{1}{\rho \sin\theta };E_{0}^{3}=\frac{a}{\sqrt{\Delta }\rho },E_{3}^{0}=\frac{a}{\rho }\sin\theta$	${\left(\sqrt{\Delta }\rho \sin\theta \right)}^{1/2}$

${}^{\left(1\right)}\;N\left(r,\theta \right)$ is f(r,θ) for spherically symmetric spacetimes and to f(r,θ)g(r,θ) in the case of stationary rotating Kerr spacetime. ${}^{\left(2\right)}\;F=\sqrt{1-kr^{2}},k=\pm 1,a$ universe size, *r* dimensionless parameter. ${}^{\left(3\right)}\;F=\left(1-\frac{2GM}{r}\right),G$ gravitation constant, *M* mass. ${}^{\left(4\right)}$$\Delta =r^{2}+a^{2}-2Mr,\rho^{2}=r^{2}+a^{2}\cos^{2}\theta ;$a=J/Mc,J angular momentum.

## Appendix A

### Appendix A.1. Properties of the Gamma Matrices

The Gamma matrices $\gamma^{\mu }$ form presentations of bi-quaternions; they factorize the d’Alembertian operator and have eigenvalues +1, and −1 only. This is a reflection of the fact that the subsidiary conditions are encoded in the gamma matrices [1]. The gamma matrices satisfy the following relations:

(A1) $${\left(\gamma^{a}\right)}^{†}=\gamma^{a};{\left(\gamma^{a}\right)}^{2}=\gamma^{0}$$

(A2) $$\left\{\gamma^{a},\gamma^{b}\right\}=2I^{\left(0\right)}\delta^{ab};a,b=0,1,2,3,$$

(A3) $$\left[\gamma^{a},\gamma^{b}\right]=i\varepsilon^{abc}\gamma^{c};a,b,c=0,1,2,3,$$

(A4) $$trace(\gamma^{a})=0;a=1,2,3.$$

The gamma matrices above carry tangent space indices so that they maintain a flat spacetime form. The spinor representations of the Lorentz generators are commutators of the gamma matrices, an observation that simplifies the calculation of the affine connection for the wave function Φ.

Lemma: For any two three vectors A and B,

(A5) $$\left(\gamma \cdot A\right)\left(\gamma \cdot B\right)=A\cdot B+\frac{i}{2}\gamma \cdot A\times B.$$

Proof is as follows:

$$\left(\gamma^{i}A_{i}\right)\left(\gamma^{j}B_{j}\right)=\gamma^{i}\gamma^{j}A_{i}B_{j}$$

(A6) $$=\delta_{ij}{\left(\gamma^{i}\right)}^{2}A_{i}B_{j}+\frac{i}{2}\epsilon^{ijk}\gamma_{k}A_{i}B_{j}=\gamma^{0}A_{i}B_{i}+\frac{i}{2}\gamma_{k}{\left(A\times B\right)}^{k},$$

where we have used Equations (A1)–(A4).

### Appendix A.2. Conserved Current

By factorizing the d’Alembertian operator, we obtain two equations (see Ref. [13] for more details):

(A7) $$\gamma^{\nu }\left(x\right)\left(\partial_{\nu }+\Omega_{\nu }\right)\Phi \left(x\right)=0,$$

(A8) $$\gamma^{\mu }\left(x\right)\left(\partial_{\mu }+\Omega_{\mu }^{*}\right)\Phi^{*}\left(x\right)=0,$$

where $\Phi^{*}$ is the complex conjugate of Φ, $\Omega_{\mu }\left(x\right)$, and $\Omega_{\mu }^{*}\left(x\right)$ are the connection coefficients for the functions $\Phi \left(x\right)$ and $\Phi^{*}\left(x\right)$. Here, we note that $\Omega \left(x\right)=-\Omega^{*}\left(x\right)$. We use the equations above to prove our claim for the conserved current. We multiply Equation (A7) by the Hermitian conjugate $\Phi^{H}$ and Equation (A8) by the transposed $\Phi^{T}$ of Φ, and sum both equations to obtain the following:

(A9) $$E_{a}^{\nu }\psi_{1}^{H}\gamma^{a}\partial_{\nu }\psi_{1}+\cdot \cdot +E_{a}^{\nu }\psi_{\left(4s\right)}^{H}\gamma^{a}\partial_{\nu }\psi_{\left(4s\right)}+E_{a}^{\nu }\psi_{1}\gamma^{a}\partial_{\nu }\psi_{1}^{H}+\cdot \cdot +E_{a}^{\nu }\psi_{\left(4s\right)}\gamma^{a}\partial_{\nu }\psi_{\left(4s\right)}^{H}=0.$$

Now, we can sum pairs of terms, such as the following:

(A10) $$E_{a}^{\mu }\psi_{n}^{H}\gamma^{a}\partial_{\mu }\psi_{n}+E_{a}^{\mu }\psi_{n}\gamma^{a}\partial_{\mu }\psi_{n}^{H}=\partial_{a}\left(\psi_{n}^{H}\gamma^{a}\psi_{n}\right).$$

Inserting Equation (A10) in Equation (A9) yields the following:

(A11) $$\partial_{0}\left(\Phi^{H}\Phi \right)+\partial_{k}\left(\Phi^{H}\gamma^{k}\Phi \right)=0;k=1,2,3.$$

This explains intuitively why the conserved current does not depend on the spin connection. Note that $\Phi^{H}\Phi$ is positive definite and is interpreted as the probability density.

### Appendix A.3. Commuting Variable

In what follows, we show that the total angular momentum J=L+S, $J^{2}$, $J_{3}$, κ=2γ·L−1 and parity P form a complete set of commuting observables. To show that, we recall that the Hamiltonian of the free field and the $i^{th}$ component of the orbital and spin angular momentum are given by the following:

(A12) $$H_{0}=-i\gamma^{l}\partial_{l},$$

(A13) $$L_{i}=-i\epsilon^{ijk}x_{j}\partial_{k},$$

and,

(A14) $$S_{i}=\epsilon^{ijk}\gamma_{j}\partial_{k}.$$

Then, by simple algebraic manipulations, one finds the following:

(A15) $$\left[L_{i},H_{0}\right]=+\epsilon^{ijk}\gamma_{j}\partial_{k},$$

and,

(A16) $$\left[S_{i},H_{0}\right]=-\epsilon^{ijk}\gamma_{j}\partial_{k}.$$

Then, from the expressions of Equation (A15) and (A16), the *i*th component of the total angular momentum commutes with the Hamiltonian such that the following holds:

(A17) $$\left[J_{3},H_{0}\right]=0,$$

(A18) $$\left[J^{2},H_{0}\right]=0.$$

The operator K=2γ·L−1 also commutes with $J_{i}$ since the following holds:

(A19) $$\left[L_{i},K\right]=\left[L_{i},2\gamma \cdot L\right]=2\left(L_{i}\gamma^{j}L_{j}-\gamma^{j}L_{j}L_{i}\right)=2i\gamma^{j}\epsilon^{ijk}L_{k},$$

(A20) $$\left[S_{i},K\right]=\left[\gamma_{i},2\gamma \cdot L\right]=2\left(\gamma_{i}\gamma^{j}L_{j}-\gamma^{j}L_{j}\gamma_{i}\right)=2\left[\gamma^{i},\gamma^{j}\right]L_{j}=2i\epsilon^{ikj}\gamma_{j}L_{k}.$$

These two expressions above sum to zero; hence, we have the following:

(A21) $$[J_{i},K]=0,$$

Now we show that the parity operator commutes with the Hamiltonian. By definition, we have the following:

(A22) $$P=\beta P;\;\;\;\beta =\left(\begin{array}{cc} I_{2s} & 0\\ 0 & -I_{2s} \end{array}\right),$$

where P and *P* are the parity operators acting on the spinors and on coordinates, respectively. Indeed, the following holds:

$$\left[P,H_{0}\right]=\left[\beta P,-i\gamma^{k}\partial_{k}\right]=-i\beta P\gamma^{k}\partial_{k}+\gamma^{k}\partial_{k}i\beta P=-i\beta \gamma^{k}P\partial_{k}+i\gamma^{k}\beta \partial_{k}P$$

(A23) $$=-i\beta P\gamma^{k}\left(-\partial_{k}P\right)+i\gamma^{k}\beta \partial_{k}P=i\left\{\beta ,\gamma^{k}\right\}\partial_{k}P=0.$$

We have used the fact that *P* does not depend on the coordinates and, therefore, $\partial_{k}P=0.$ Furthermore, the parity operator commutes with $J_{i}$ since $\left[P,J_{i}\right]=\beta \left[P,J_{i}\right]$. The coordinate parity operator *P* commutes with $J_{i}$ since the angular momentum is a pseudovector.

$$\left[K,H_{0}\right]=\left[\gamma^{i}L_{\ddot{ı}},-i\gamma^{k}\partial_{k}\right]=-i\beta P\gamma^{k}\partial_{k}+\gamma^{k}\partial_{k}i\beta P=-i\beta \gamma^{k}P\partial_{k}+i\gamma^{k}\beta \partial_{k}P$$

$\left[H_{0},K\right]=\left[-i\;\gamma \cdot \hat{r}\left(\partial_{r}+\frac{1}{r}-\frac{1}{r}K\right),K\right]$

=−i $\gamma \cdot \hat{r}\left(\partial_{r}+\frac{1}{r}-\frac{1}{r}K\right)K+iK$ $\gamma \cdot \hat{r}\left(\partial_{r}+\frac{1}{r}-\frac{1}{r}K\right)$

$=\left[-i\;\gamma \cdot \hat{r}\left(\partial_{r}+\frac{1}{r}\right),K\right]+\left[i\;\frac{1}{r}\gamma \cdot \hat{r}K,K\right]$

$\;\;\;\;\;\;\;\;\;\;=-i\left(\partial_{r}+\frac{1}{r}\right)\left[\gamma \cdot \hat{r},K\right]+i\frac{1}{r}\left[\gamma \cdot \hat{r}K,K\right]$

$\;\;\;\;\;\;\;\;\;\;=-i\left(\partial_{r}+\frac{1}{r}\right)\left[\gamma \cdot \hat{r},\gamma \cdot L\right]+i$ $\frac{1}{r}\left[\gamma \cdot \hat{r}\gamma \cdot L,\gamma \cdot L\right]$

$\;\;\;\;\;\;\;\;\;\;=-i\left(\partial_{r}+\frac{1}{r}\right)\left(\gamma \cdot \hat{r}\gamma \cdot L-\gamma \cdot L\gamma \cdot \hat{r}\right)+i$ $\frac{1}{r}\left(\gamma \cdot \hat{r}\gamma \cdot L\gamma \cdot L-\gamma \cdot L\gamma \cdot \hat{r}\gamma \cdot L\right)$

$\;\;\;\;\;\;\;\;\;\;=-i\left(\partial_{r}+\frac{1}{r}\right)\left(\gamma \cdot \hat{r}\gamma \cdot L-\gamma \cdot L\gamma \cdot \hat{r}\right)+i$ $\frac{1}{r}\left(\gamma \cdot \hat{r}\gamma \cdot L-\gamma \cdot L\gamma \cdot \hat{r}\right)\gamma \cdot L$

Now, the anti-commutator in the parenthesis vanishes. Using the identity above, we have the following:

$$\left(\gamma \cdot \hat{r}\gamma \cdot L-\gamma \cdot L\gamma \cdot \hat{r}\right)=\left(\hat{r}\cdot L+i\frac{1}{2}\gamma \cdot \hat{r}\times L-L\cdot \hat{r}-i\frac{1}{2}\gamma \cdot L\times \hat{r}\right)=0.$$

To conclude, $H_{0},J^{2},J_{3},K$ and *P* form a complete set of commuting operators.
